# Three-Dimensional Optical Trapping of a Plasmonic Nanoparticle using Low Numerical Aperture Optical Tweezers

**DOI:** 10.1038/srep08106

**Published:** 2015-01-29

**Authors:** Oto Brzobohatý, Martin Šiler, Jan Trojek, Lukáš Chvátal, Vítězslav Karásek, Aleš Paták, Zuzana Pokorná, Filip Mika, Pavel Zemánek

**Affiliations:** 1 ASCR, Institute of Scientific Instruments, Královopolská 147, 612 64 Brno, Czech Republic

## Abstract

It was previously believed that larger metal nanoparticles behave as tiny mirrors that are pushed by the light beam radiative force along the direction of beam propagation, without a chance to be confined. However, several groups have recently reported successful optical trapping of gold and silver particles as large as 250 nm. We offer a possible explanation based on the fact that metal nanoparticles naturally occur in various non-spherical shapes and their optical properties differ significantly due to changes in localized plasmon excitation. We demonstrate experimentally and support theoretically three-dimensional confinement of large gold nanoparticles in an optical trap based on very low numerical aperture optics. We showed theoretically that the unique properties of gold nanoprisms allow an increase of trapping force by an order of magnitude at certain aspect ratios. These results pave the way to spatial manipulation of plasmonic nanoparticles using an optical fibre, with interesting applications in biology and medicine.

Noble metal nanoparticles (NPs) have attracted increased attention in recent years due to various applications of resonant collective oscillations of free electrons excited with light (*i.e.* plasmon resonance). In contrast to bulk metal materials, where this plasmon resonance frequency depends only on the free electron number density, the optical response of gold and silver NPs can be tuned over the visible and near-infrared spectral region by the size and shape^1^ of the NP. Most applications of metal NPs are based on the substantial heating they undergo when illuminated near the plasmon resonance. Such nanosources of heat find unique applications in plasmonic photothermal therapy and delivery, nanosurgery, photothermal and photoacoustic imaging, plasmon-assisted nanochemistry or optofluidics^2^,^3^,^4^. Precise and remote placement and orientation of NPs inside cells or tissue would provide another degree of control for these applications. Concerning NP applications in biology, gold is the most frequently used metal due to its several unique properties. First of all, gold is not toxic for cells. Additionally, the plasmon resonance can be excited by visible or NIR wavelengths which penetrate deep into the tissue. Finally, gold surfaces are resistant to oxidation and can be functionalized by many chemical compounds. However, gold is nonmagnetic and therefore the straightforward manipulation and separation techniques based on magnetic field can not be applied. On the other hand, laser beam can be used not only for plasmon excitation but also for manipulation with NPs.

A single focused laser beam – optical tweezers – represents the most frequently used arrangement which provides three-dimensional (3D) contact-less manipulation with dielectric objects or living cells ranging in size from tens of nanometers to tens of micrometers^5^. Applications of optical tweezers in many branches of physics, chemistry and biology led to the development of variety of techniques that revolutionized these fields^6^,^7^. Svoboda and Block demonstrated the first three-dimensional trapping of absorbing Au NP^8^ and reported about 7× stronger trapping force for Au NP of diameter 36 nm compared to a polystyrene sphere of similar diameter (38 nm). They pointed out that this was caused by the approximately 7× larger magnitude of polarizability of the gold NP asocciated with its absorption. Further experiments demonstrated 3D trapping of gold and silver nanospheres and nanorods of sizes between 20 to 250 nm^9^,^10^,^11^,^12^. Mostly, infrared trapping wavelengths were preferred since they were far from the NP plasmon resonance where the NP absorption and consequent heating was suppressed^13^,^14^,^15^.

The longitudinal equilibrium position of an optically trapped NP is a result of the balance between the *gradient force* (pulling the NP towards the beam focus) and the *scattering force* (pushing the NP in the direction of beam propagation)^16^,^17^. Hence an increase of absorption or scattering causes an increase of the scattering force and the consequent release of the NP from the trap. Thus the optical resonance properties of plasmonic NPs determine our ability to optically trap and manipulate them. For instance, three-dimensional optical trapping of metal NPs at wavelengths close to the plasmon resonance is very limited. Recent studies have shown that the gradient force can change its sign depending on the trapping wavelength and can either attract the NPs towards the high-intensity beam centre or repel them out of the beam^10^,^18^,^19^,^20^. Such NP repulsion or attraction has been experimentally demonstrated^10^,^21^ by tuning the trapping laser wavelength below or above the plasmon resonance wavelength, respectively. Moreover, Toussaint *et al.*^22^ have shown that in the Rayleigh regime the gradient force acting upon core-shell nanorods or particles of bi-pyramidal shape can be enhanced when the trapping laser wavelength is slightly red-detuned from the plasmon resonance. Messina *et al.*^23^ have demonstrated trapping enhancement when gold NPs are aggregated into a nanostructure with controllable extinction properties.

Controlling the shape of a plasmonic NP represents the most powerful means of tailoring and fine-tuning not only its optical resonance properties but also optical forces exerted on it when the NP is irradiated by light. Here we demonstrate experimentally and explain theoretically the optical trapping of gold NPs of various shapes with sizes between 20 and 250 nm in optical tweezers where a laser beam is focused by an aspherical lens with low numerical aperture (NA) varying between 0.37 and 0.2, *i.e.* with a diameter of the beam focus between 1.8 *μ*m and 3.4 *μ*m. Such an extremely low NA offers the possibility to bring the technique of optical manipulation of NPs into the area of biophotonic endoscopy, done with multimode fibres^24^,^25^, or photonic crystal fibres^26^, where higher values of NA cannot be achieved.

## Results

### Trapping geometry

**Figure 1:**
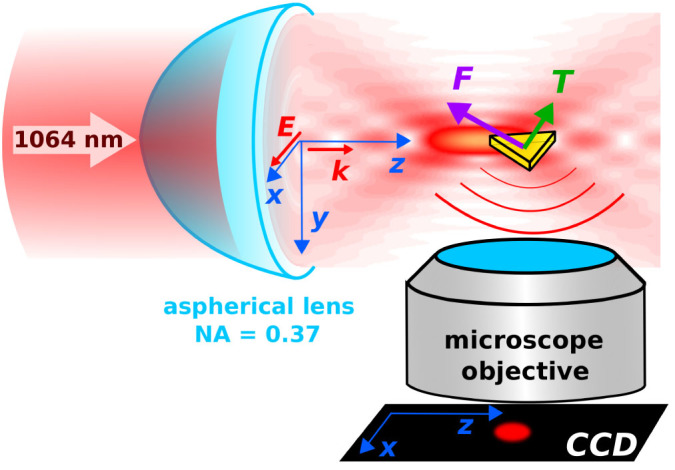
Trapping geometry. A naturally shaped NP (yellow triangular prism) is trapped laterally on the optical axis and longitudinally slightly behind the beam focus (bright yellow spot). The longitudinal position of the NP strongly depends on its orientation with respect to the direction of beam polarization and propagation. The objective and the CCD camera were placed perpendicularly to the direction of beam propagation and image the NP as a bright spot on the dark background.

Optical trapping was investigated in an experimental setup (see Methods for details) with a horizontally propagating trapping beam as illustrated in Figure 1. The setup employed the spatial light modulator (SLM) as the key optical element enabling the control of trapping beam properties^27^, *e.g.* NA of the trapping beam. Furthermore, the SLM allows in situ wavefront optimization^28^, eliminating aberrations introduced in the optical pathway. The beam emitted from a 1064 nm infrared laser source was focused by an aspherical lens of maximum numerical aperture NA = 0.37 instead of a microscope objective with a high NA as is usual in common optical tweezers. This provided us with an aberration-free laser beam, with beam diameter in the range 1.8–3.4 *μ*m, which appeared to be very important in our trapping application (see Methods for details). A diluted colloidal suspension of NPs (British Biocell) was placed into a glass capillary and illuminated with the focused laser beam. A single NP was trapped in the beam focus and observed from the side employing a long-working-distance microscope objective. It has to be noted that the commercial NPs were often nominally spherical. We will show that this is far from reality and that the typical commercial colloidal suspension contains rather non-spherical NPs.

### Measurement of trap properties

**Figure 2:**
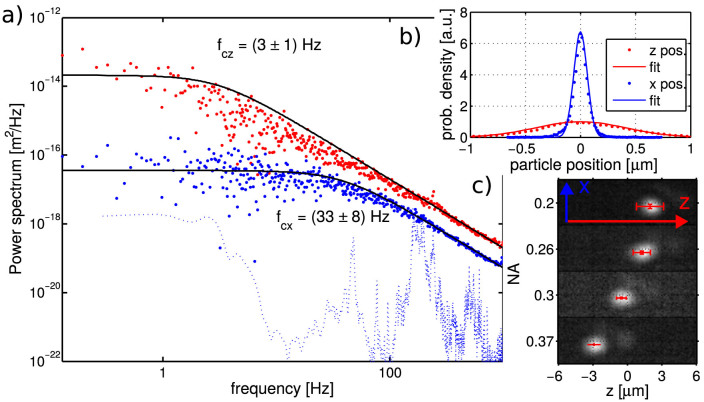
An example of quantitative experimental results for Au NP of size 250 nm. (a) The power spectral density of NP positions along *x* (red) and *z* (blue) axis and the black curves denote the fit of the data with Lorentzian function^29^,^30^,^31^. The dotted curve shows the power spectral density profile for the trapping beam directed on the camera without a trapped NP. (b) The probability density of NP occurrence along *x* (blue) and *z* (red) axis. 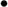 and full curve denote measurement and its fit with Gaussian density distribution 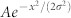, respectively. (*σ_x_* = 60 nm and *σ_z_* = 410 nm) (c) Images of a NP of size 250 nm for different NA of the trapping beam and corresponding shift of its longitudinal stable position plotted together with NP position standard deviation (red errorbars). For more details see Methods.

The trapped NP was observed as a bright spot on a dark background because the CCD camera was oriented perpendicularly to the beam propagation and collected only the light scattered by the NP. In contrast to the common optical tweezers, this side-view observation enables us to determine the number of NPs along the longitudinal axis and the lateral and longitudinal trap properties directly from the CCD images. Even though the spot size on the CCD does not correspond to the real size of the NP, its motion on the CCD follows the motion of the NP in the trap and thus can be used to quantify the properties of the optical trap^29^ (*e.g.* trap stiffness and potential profile, see Methods for details). Figure 2 shows an image of a single trapped NP (size 250 nm) together with the probability density of the NP positions and the corresponding power spectral density. These results show that the NP is localized within an area of ±120 nm laterally and ±820 nm longitudinally (with the probability 95.4%). Power spectral densities fit well the Lorentzian dependence and the signal for the trapped NP is above the background noise, as the dotted curve demonstrates. These measurements and analyses were also repeated for NPs of sizes from 50 nm up to 250 nm. In contrast to the previously published results, here we report the first 3D confinement of a gold NP as large as 250 nm in a moderately focused laser beam of diameter 1.8–3.4 *μ*m (NA = 0.37 − 0.2). The lateral confinement is not as surprising as the longitudinal one which is not predicted by the theory for spherical NPs illuminated by a beam of such a low NA.

### Basic theoretical background of a gold NP in an optical trap

The NP behaviour along the longitudinal direction (see Figure 1) can be qualitatively understood using the terminology of the gradient and scattering optical forces^7^,^16^,^17^,^32^ even though this is fully valid only for tiny absorbing NPs. The longitudinal component of the optical force acting upon a NP placed on the beam axis can be written as (see Methods for details): 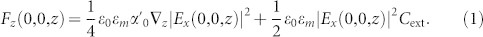
The first term is proportional to the real part of the NP polarizability 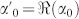 and the gradient of the optical intensity. Therefore it is known as *the gradient force* and pushes the NP toward the high intensity part of the beam (*i.e.* beam focus) if 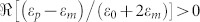. If the particle is located behind the beam focus, this force is negative and pulls the NP back to the beam focus^7^. The second term is known as *the scattering force*, it is always positive and proportional to the NP absorption and scattering cross sections (under the approximation considered here). Therefore, it pushes the NP along the direction of beam propagation. The NP can be optically trapped if the longitudinal force is equal to zero and has a negative slope for some longitudinal position, *i.e.* here the gradient force balances the scattering force. The gradient force and the part of the scattering force originating in absorption are proportional to *a*^3^ and they scale the same way with increasing particle radius *a*. However, the part of the scattering force coming from the scattering of light is proportional to *a*^6^, therefore, the equilibrium position (optical trap) for a larger NP is pushed along the direction of beam propagation away from the beam focus, see Figure 2c. Eventually the gradient force cannot compensate the scattering one, the optical trap disappears, and the NP is released and pushed along the direction of beam propagation mainly by the scattering force. Besides particle size, the force balance is influenced by the trapping wavelength because the permittivity of a metal NP exhibits a strong spectral dependence near the plasmon resonance. Consequently the wavelength dependent increment of absorption and scattering cross sections will lead to the same behaviour as described above for larger NPs, *i.e.* the gradient force will be weaker than the scattering one and the NP will be propelled along the direction of beam propagation.

### Spherical NP

**Figure 3:**
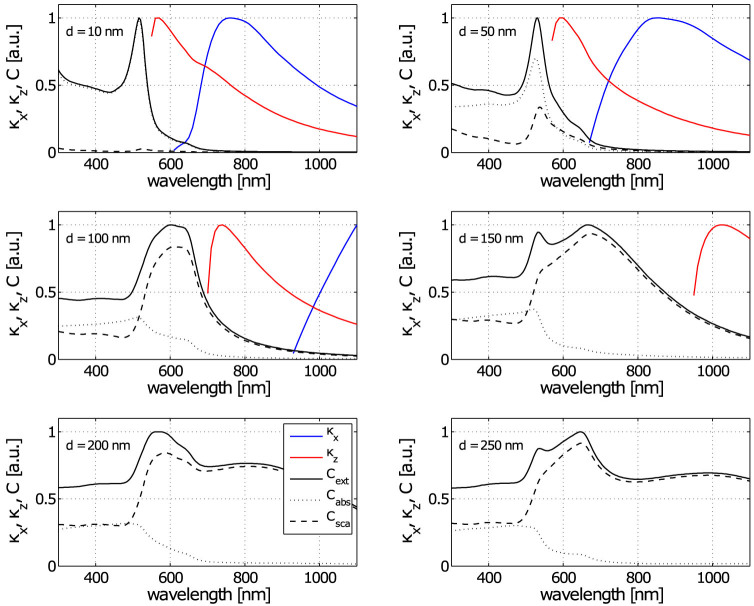
Extinction cross sections and trapping stiffness calculated for gold nanosphere. Absorption, scattering and extinction cross sections calculated for gold nanospheres of various diameter *d* reveal their spectral broadening and red-shift of their maximum for larger spheres. The optical trap stiffness calculated for the same nanospheres placed on the beam axis of a single focused beam of numerical aperture NA = 0.37 is added to the plots, too. Note that gold nanospheres of diameter larger than 120 nm cannot be optically trapped and therefore the stiffness curves are missing. The stiffnesses and extinction cross sections are normalized to their maximum value; absorption and scattering cross sections are normalized to the maximum of *C*_ext_ for each particle size.

Like most previous studies, we start our analyses with the spherical Au NPs. Figure 3 presents the spectral dependencies of absorption, scattering, and extinction cross sections together with trap stiffnesses obtained from generalized Lorenz-Mie theory^33^ for six different NP diameters. We used the value of NA = 0.37 of the trapping beam, the same as in our experiments (see Methods for description of the trapping beam). Direct comparison of cross sections with lateral (*κ_x_*, blue curve) and longitudinal (*κ_z_*, red curve) trap stiffnesses reveals spectral regions where the absorption and scattering become dominant and shows how they influence the NP longitudinal confinement following Eq. 1. The trap stiffnesses are maximal for trapping wavelengths red-detuned from the plasmon resonance which is determined by the maximal value of the extinction cross section *C*_ext_. This theoretically verifies the experimentally observed concept of plasmon-resonance-based optical trapping^10^,^22^,^23^ even for larger metal particles.

The results support the conclusion that light absorption is the leading mechanism behind the scattering force for gold spheres with diameters *d* < 50 nm because the scattering cross section is negligible here. In contrast, light scattering becomes the leading source of the scattering force for gold spheres with diameters *d* > 50 nm and, at wavelengths longer than about 550 nm, is responsible for missing optical traps. This trend is also enhanced due to the shift and broadening of the plasmon resonance peak towards longer wavelengths for larger NPs (see Fig. 3, *d* ≥ 150 nm). Furthermore, for larger NPs (*d* = 150 nm) the scattering force is so strong that the longitudinal stable position of a NP is shifted into an axial local intensity minimum located behind the beam focus (see spatial intensity profile in Figure 1 and Refs 34, 35). Thus, the lateral force acts away from the optical axis and the NP cannot be stably trapped.

However, numerical results in Figure 3 are in contradiction with our experimental observations and also with previous observation of Hansen *et al.*^9^. They observed that gold NPs can be stably optically trapped in high NA optical tweezers even if their diameters are larger than 170 nm. Saija *et al.*^36^ tried to compare their theoretical simulations with experimental trapping stiffnesses measured by Hansen *et al.* showing very similar discrepancy between theory and experiment. They too considered spherical NPs in their model and tried to explain the experimental observation by suggesting that a steam nanobubble is formed around the trapped nanosphere. Such nanobubble formation was indeed observed experimentally^37^,^38^ but recent studies also demonstrated that gold NPs can be heated up to the melting^14^,^15^ temperature without any nanobubble appearing. Obviously, the mechanism behind the steam nanobubble formation and its stability is not fully understood and is still under intense investigation.

### Possible thermal effects

We looked for the origin of the discrepancy of theory and experiment. We focused first on the influence of the hydrodynamic drag and thermophoretic forces^40^ acting upon the NP which come from the thermal fluid flow induced by the absorption of the laser energy in the surrounding medium and temperature gradients around the NP, respectively. We exchanged H_2_O by D_2_O having about 10× lower coefficient of linear absorption at 1064 nm^41^ and we repeated the experiments. However, we have not observed any difference in the NP behavior, *i.e.* the large NPs were again spatially confined. Therefore we excluded the principle influence of these thermal effects.

### Naturally shaped NP

**Figure 4:**
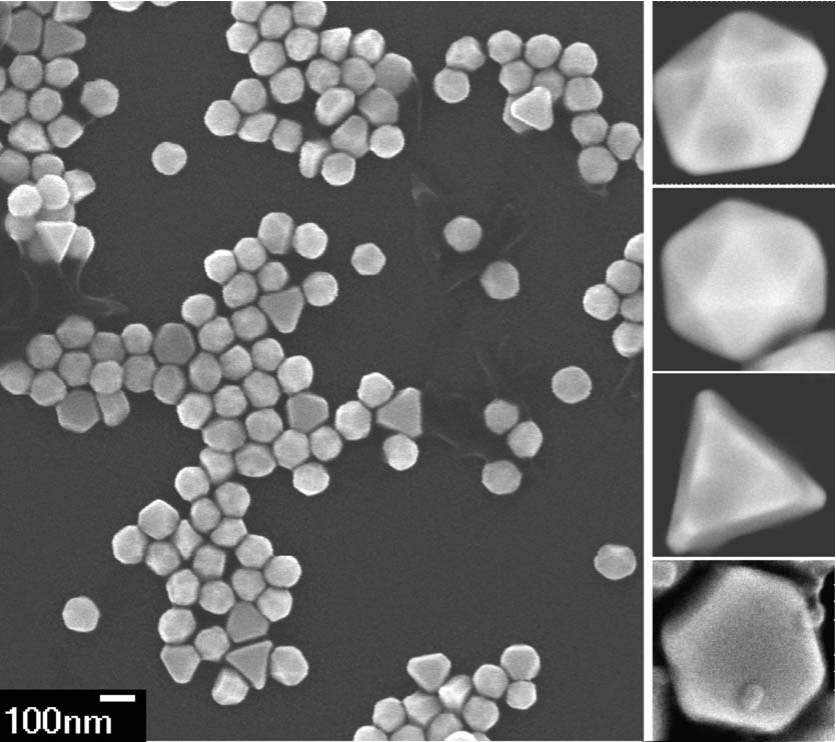
Gold NPs observed by scanning electron microscope. Two scanning electron microscopes (JEOL JSM-6700F and FEI Magellan 400) were used to study a shape of NPs (100 nm British Biocell). Right-hand column shows detailed images of various particle shapes naturally contained in samples of NPs larger than 50 nm: decahedron, icosahedron, triangular and hexagonal prisms.

**Figure 5:**
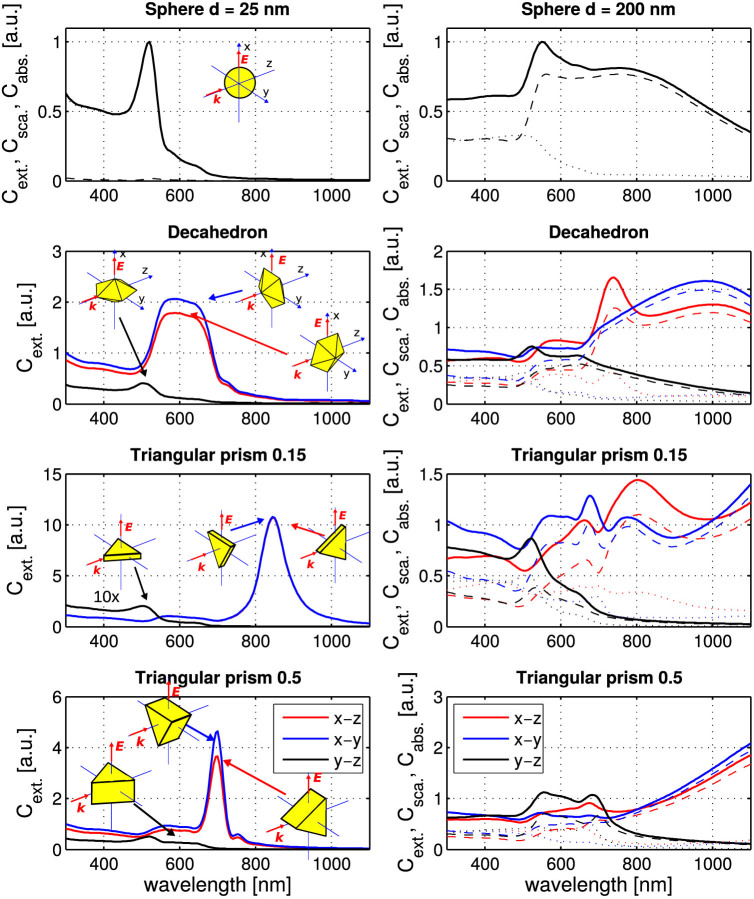
Extinction, scattering and absorption cross sections calculated for gold NPs. Extinction (full lines), scattering (dashed lines) and absorption cross sections (dotted lines) were calculated by coupled dipole method for Au nanosphere, decahedron, and triangular prisms with aspect ratios 0.15 and 0.5 (see Methods for definition). The spectra were calculated for three orientations of the NP relative to the orientation of the incident plane wave polarization (*x* axis) and propagation axis (*z* axis). Extinction spectra for each NP are plotted relative to the maximal value of the extinction cross section of the sphere of the same volume as the NP. *Left:* NPs having the same volume as a sphere with diameter 25 nm. *C*_ext_ and *C*_abs_ curves overlap and thus only *C*_ext_ is shown. Rather flat NPs, as the triangular prisms with the aspect ratio equal to 0.15, exhibit a strong red-shift of extinction maximum for NPs oriented in the *xz* and *xy* plane due to increased charge separation in the NP. Note, that in the case of the triangular prism, where the aspect ratio equals 0.15, the blue and the red lines overlap. *Right:* NPs of the same volume as a sphere with diameter 200 nm. If the studied NP is oriented in the *yz* plane, the lowest extinction cross section is obtained for wavelengths *λ* > 700 nm, *i.e.* the longitudinal scattering force is suppressed according to Eq. 1.

Finally, we came to the conclusion that the only parameter that has not been taken into account, is the natural non-spherical shape of the NP. The scanning electron microscope images of the NP sample are shown in Figure 4. The natural shapes of Au NPs can be described as decahedrons, icosahedrons, hexagonal and triangular prisms^1^,^42^. In the case of a non-spherical NP, its cross sections depend on the NP orientation with respect to direction of beam polarization and propagation. Figure 5 shows the spectral profiles of cross sections for three principal orientations of the NP with respect to the incident plane wave. The strongest interaction of investigated NPs with the incident field occurs if the NP is oriented parallel to *xz* and *xy* plane when the longitudinal red-shifted plasmon modes are excited. On the other hand, the scattering cross sections are much smaller if the flat NP is oriented perpendicular to the polarization direction (*i.e.* in *yz* plane). Consequently, the value of *C*_ext_ in Eq. 1 and the longitudinal ‘pushing' scattering force depend strongly on the orientation of the non-spherical NP in the optical trap.

**Figure 6:**
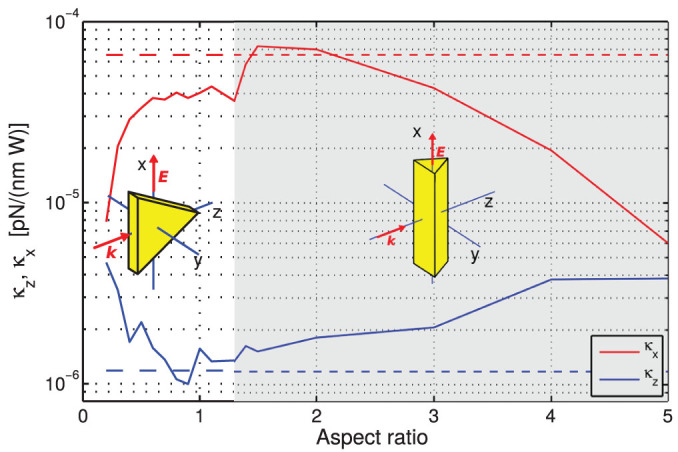
Enhancement of trap stiffnesses by tuning the aspect ratio of a triangular prism. Longitudinal (*κ_z_*) and lateral (*κ_x_*) stiffnesses of a stably trapped and oriented triangular prism of various aspect ratios were calculated (see Methods for definition). Its volume is fixed and equal to the volume of a nanosphere of diameter 20 nm. Trapping stiffnesses of such nanospheres are plotted by dashed lines. We used CDM and considered single focused beam with the following parameters: vacuum wavelength *λ*_vac_ = 1064 nm, NA = 0.37, beam diameter 1.8 *μ*m, incident power 1 W. Different stable orientations of the nanoprism are highlighted by the white and gray background.

However, beside the optical force, the light also exerts a torque on non-spherical NPs^20^,^43^,^44^ and thus the NP stable orientation in the optical trap is the key parameter for the 3D NP trapping. Using coupled dipoles method (CDM) we calculated optical forces and torques acting upon gold triangular prisms and decahedrons in various positions and orientations. Stable orientations and positions were determined from zero values of torques and forces with negative slopes in angular inclinations and spatial displacements from the stable geometry. Figure 6 illustrates how the stable orientation and trap stiffnesses are changed for a gold triangular prism when its aspect ratio (see Methods for definition) increases. NPs of aspect ratio smaller than 0.2 can not be trapped in 3D under the considered conditions. These results reveal that the lateral trap stiffness *κ_x_* of the triangular nanoprism is mostly weaker compared to spherical NP. For some aspect ratios the value of *κ_x_* drops even by one order of magnitude. In contrast, longitudinal stiffness of the triangular nanoprism is stronger for most aspect ratios compared to the spherical NP. This result indicates that a non-spherical NP can be longitudinally trapped more strongly when compared to the usually considered nanospheres.

### Comparison of experimental and theoretical trap stiffnesses

**Figure 7:**
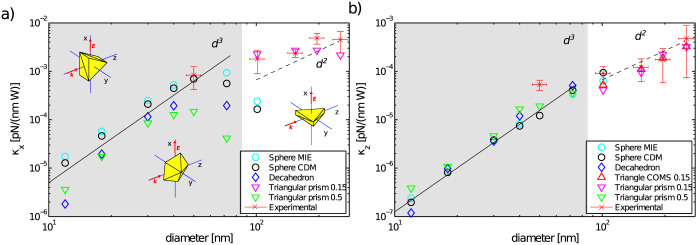
Comparison of experimental and theoretical trap stiffnesses. The measured and calculated (a) lateral (*κ_x_*) and (b) longitudinal (*κ_z_*) trap stiffnesses of an optically trapped NP of various sizes and shapes are compared. The single focused beam had the following parameters: vacuum wavelength *λ*_vac_ = 1064 nm, NA = 0.37, beam diameter in the beam focus 1.8 *μ*m, incident power is recalculated to 1 W. The following NP shapes were considered: spheres calculated by Mie and CDM, decahedrons calculated by CDM, triangular nanoprisms with two aspect ratios (0.15 and 0.5) calculated by CDM and Comsol (aspect ratio 0.15 only). Experimental results are marked with crosses together with estimated standard deviations of NP sizes and trap stiffnesses. The grey and white backgrounds denote the calculated stable orientations of NPs. Note, that the small thinner triangular nanoprisms *d* < 100 nm (aspect ratio 0.15, violet triangles) are not stably trapped if their orientation is parallel to the direction of beam polarization and therefore the violet triangles are missing in the grey area.

Figure 7 compares the theoretical trap stiffnesses for several sizes of naturally shaped NPs stably trapped and oriented in the optical trap. Both numerical methods gave comparable results for spherical and thin triangular prism NPs. This verifies the correctness of the CDM results. The plots are split into two parts (denoted by the background colour) according to the stable orientation of the NPs, obtained from the theoretically calculated torque. Smaller thick triangular nanoprisms (aspect ratio 0.5, green triangles) and decahedrons (blue diamonds) are oriented with their longer axis parallel to the beam polarization. Their trapping stiffnesses are depicted in the grey area. As the particle size increases above 100 nm, the scattering force on these nanoparticles gets stronger and stable trapping vanishes (white area in Figure 7). However, our calculations showed that thinner triangular prisms (aspect ratio 0.15, violet triangles) reorient themselves with their longer axis perpendicular to the direction of the beam polarization and thus the scattering force acting on them dropped (see Figure 5) and stable trapping is restored. The trapping stiffnesses are depicted as violet triangles in the white area of Figure 7. The aspect ratio of gold nanoprisms is a crucial parameter because it determines stable orientation of nanoprisms in the optical trap and also their overall trapping stability. We expect similar behaviour for other thin prisms, such as hexagonal ones.

In the same figure, red crosses with errorbars depict the experimental trapping stiffnesses together with estimated standard deviations of NP sizes and the trap stiffnesses (for details see Methods). Comparison of the measured and theoretical stiffnesses indicates a very good quantitative correspondence. We tried to characterize our samples employing the scanning electron microscopy (SEM). The size distributions of icosahedrons (which are most similar to the spherical nanoparticles) are in good agreement with the data provided by the manufacturer, *i.e.* standard deviation is about 8%. However, the SEM revealed wide variation of the aspect ratios of triangular and hexagonal prisms presented in the samples, which consequently leads to the large variation in the experimentally determined trapping stiffness. Unfortunately, it was not possible to identify the shape and orientation of the NP in the trap from the CCD images, but the agreement between the measured and calculated stiffness for thin triangular nanoprism encourages us to conclude that the natural shape of the NP is responsible for 3D optical trapping of large Au NPs.

Since the trapping power used in the measurements varied between 100 or 200 mW in the sample plane, we normalized the experimental stiffnesses to the trapping power of 1 W used in the theoretical calculations, assuming direct proportionality between trap stiffness and trapping power.

The incoming electromagnetic field is shielded by free electrons inside the metal NP and thus the certain penetration depth exists – so called skindepth. This behaviour is, in our theoretical model, fully described by imaginary part of the refractive index and the typical magnitude of the skindepth of the gold NPs is about 50 nm. The gold NPs smaller than *d* < 100 nm interact with the trapping laser beam by their entire volume and thus the trapping force scales with *d*^3^. On the other hand bigger particles interact effectively just by surface layers and therefore the trapping force scales with *d*^2^. The full and dashed black lines in Figure 7 guide eyes for *d*^3^ and *d*^2^ force dependence, respectively. Note, that this behaviour is fulfilled only for spherical NPs, in the case of thin nanoprism the effective layer is larger. The change in the slope is, in our case, given rather by the change of stable orientation of the NPs in the optical trap and by the aspect ratio of the considered nanoprism.

## Discussion

We presented experimental results demonstrating 3D optical trapping of relatively large Au NPs in a moderately focused laser beam with a NA of less than 0.37. If one considers only spherical NPs, these observations are not supported by the generalized Lorenz-Mie theory. However, if we assume natural non-spherical shapes of Au NPs, and calculate their stable orientation in the optical trap, we obtain a very good coincidence between the trap stiffnesses determined from the experimental data and coupled dipole method for triangular prism NPs. This supports our conclusion that the 3D trapping of larger Au NPs is caused by their non-sphericity and proper stable orientation in the trap even though they are smaller than the trapping wavelength. Based on these conclusions it seems that in our case the irradiation of metallic NPs does not lead to melting and restructuring of NPs^39^, *e.g.* gold nanorods or nanoprisms restructure into more spherical shapes and thus they change significantly their optical properties.

Although the trapping stiffness in our system is approximately 10× weaker than that reached in a common high NA optical tweezers^9^, we believe that these are promising benefits, *e.g.* the low NA system can be easily adapted using fibre systems. Thus, optical manipulation and NP heating could be easily combined with imaging, leading to flexible fibre endoscopy with many applications in biology or medicine. Other application can be found in the sorting of NPs according to their shapes. Since only NPs of a certain shape are trapped, NPs of other shapes are propelled along the direction of beam propagation and thus can be optically separated^45^,^46^.

## Methods

### Basic theory of optical forces acting upon a NP

The optical force acting upon a tiny particle placed on the axis of a beam linearly polarized along the *x* axis can be described as ref. 16

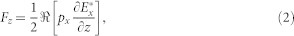

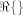
, 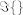, and 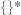 denote the real part, imaginary part, and complex-conjugated value of the quantity in the brackets, respectively. *p_x_* represents the *x*-th component of the induced dipole that satisfies *p_x_* = *ε*_0_*ε_m_αE_x_*, and *α* is the polarizability of the particle, *ε*_0_ is the permittivity of vacuum, 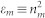 is the relative permittivity of the medium surrounding the particle. In general, polarizability has real *α*′ and imaginary *α*″ parts. The imaginary part *α*″ is associated with the particle absorption (for example in metals) and with the interaction of the induced dipole with itself through the scattered light^47^. The polarizability can be written in the form: 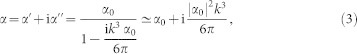
where we assumed the highly restrictive condition 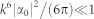, *k* = 2*πn_m_*/*λ_vac_*, and *α*_0_ can be obtained from the Lorentz-Lorenz relation: 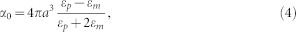
where *a* is the NP radius, 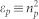 is the relative permittivity of the particle and *n_p_* is its refractive index. For an absorbing particle, *ε_p_* and consequently *α*_0_ are complex numbers. Therefore 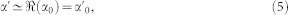

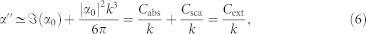
where we denoted the absorption, scattering and extinction cross section for spherical NP^48^ as *C*_abs_, *C*_sca_, and *C*_ext_, respectively. Finally, the longitudinal component of the optical force acting upon a NP located on the beam axis can be written by Eq. (1).

### Numerical methods

In the case of a spherical NP we used generalized Lorenz-Mie theory of light scattering^33^,^49^ and we calculated lateral and longitudinal optical forces acting upon a NP illuminated by a laser beam with vacuum wavelength varied from 250 to 1100 nm. We considered vectorial description of the field^20^,^50^,^51^ because it corresponds better to the experimentally observed situations^10^,^20^,^34^,^35^,^36^. We used the following parameters of the beam: numerical aperture of the focusing lens NA = 0.37, power coming through the beam focus equals to 1 W. The values of the refractive index of the NP and water for considered trapping wavelengths were taken from Palik^52^. For each wavelength we found the equilibrium position of the nanosphere and determined the lateral and longitudinal stiffnesses at this position.

In the case of non-spherical NP we extended the ADDA code^53^ based on coupled dipole method (CDM)^54^ so that we could calculate not only the cross sections but also optical torques and optical forces^55^,^56^ acting upon the NP shaped as considered above. We consequently determined the stable NP orientation and position in 3D.

### Sample preparation

The Au NPs (British Biocell, diameters 50, 100, 150, 200, 250 nm; the coefficient of variation provided by manufacture is 8%) were diluted in distilled water (or D_2_O) and put into a square glass capillary with an inner diameter of 100 *μ*m (Vitrocells 8510).

### NPs properties

We characterized sizes and shapes of NPs in our samples with SEM. We determined separately size distributions of icosahedrons, which are most similar to the sphere, and get diameters and coefficients of variation as given by the manufacturer. We also determined the size distribution of triangular and hexagonal nanoprisms presented in the samples and we observed that the diameter of the circle circumscribed to the triangular/hexagonal base is about 30% larger when compared to the average diameter of icosahedron. Unfortunately, we were not able to determine the thickness of nanoprisms and thus we were not able to determine the aspect ratios and overall volume of nanoprisms.

Following previous studies^57^,^58^,^59^ we set the aspect ratio *q* (the ratio of decahedron half height and the radius of the circle circumscribed to its pentagonal base) for natural gold decahedrons equal to 0.6. In the case of triangular prisms, we considered two values of aspect ratios (the ratio of prism height and the radius of the circle circumscribed to the triangular base) equal to *q* = 0.15 and *q* = 0.5. To compare trapping stiffnesses of the various shapes with those of the nanospheres, we considered that the NPs have the same volume as nanospheres of radius *a* = *d*/2 (their growth was simultaneous during the synthesis). Using Eqs. 7 we obtained the radius *r* of the circle circumscribed the base of a NP for a given aspect ratio *q*. 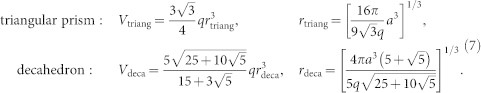


**Figure 8:**
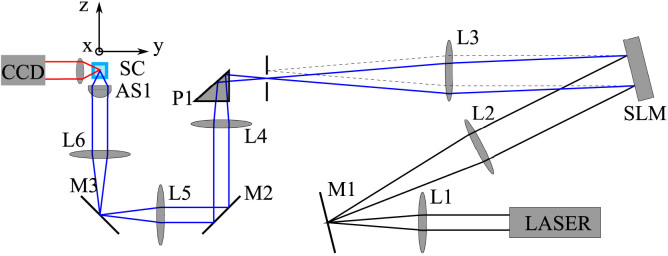
Experimental setup.

**Experimental setup** is shown and described in Figure 8. We have used Thorlabs achromatic doublets with antireflection coatings ACN254-XXX-C (L1–L6), dielectric mirrors PF10-03 (M1–M3) and aspherical lens C240TME-C with antireflection coating. A collimated Gaussian beam from IPG ILM-10-1070-LP (wavelength 1064 nm, maximal output power 10 W) is expanded by the telescope which is composed of lenses L1 (*f*_1_ = 150 mm) and L2 (*f*_2_ = 300 mm) and projected on the SLM (Hamamatsu LCOS X10468-07). Encoded phase at the SLM produces a beam in the first diffraction order in the focal plane of lens L3 (*f*_3_ = 400 mm) which is placed above the zero-order beam. NA of the trapping beam is controlled by the area of the diffraction grating imposed upon the SLM. Unwanted higher diffraction orders and the zero order are blocked by a spatial filter placed into the focal plane of L3. The transmitted beam is reflected on prism P1 and collimated by lens L4 (*f*_4_ = 200 mm). The lens L4 forms a telescope with L3 projecting the SLM plane on mirror M2. The SLM plane is imaged onto the back focal plane of an aspherical lens AS1 (*f* = 8 mm, maximal value of NA = 0.5) by a telescope consisting of lenses L5 (*f*_5_ = 100 mm) and L6 (*f*_6_ = 150 mm). AS1 focuses the beam into a square glass capillary of inner width 100 *μ*m (Vitrocells 8510) where the sample (SC) is placed. The sample is observed from the side (*xz* plane) by fast CCD camera IDT XS3.

We recorded the lateral intensity profile in the beam focal plane using the calibrated CCD camera and fitted it with a two-dimensional Gaussian profile 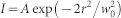. Since our theoretical beam description based on Richards and Wolf^50^,^51^ does not directly depend on the beam radius but rather on the angular or numerical aperture, we calculated the value of this aperture that corresponded to the experimentally measured beam radius. However, since we used rather low NAs, the relation *w*_0_ = *λ*/(*π* tan *θ*) for scalar Gaussian beam can be used withing 97% accuracy; *θ* is the beam angular aperture, and NA = *n_m_* sin *θ*, where *n_m_* is the medium refractive index.

### Determination of optical trap properties

Stable 3D optical trapping for all NPs sizes was observed for diameters of the trapping beam between 1.8 *μ*m and 3.4 *μ*m. However, successful 3D confinement in wider beams demanded relatively high laser power (≈500 mW) in the sample plane. Therefore, we set the beam focus diameter to 1.8 *μ*m and used a fast CCD camera to record the NP positions in the *xz* plane with a frame rate of 1000 and 2000 Hz for smaller (up to 150 nm in diameter) and larger NPs, respectively. NP positions were determined by least-square fitting of the scattered particle pattern in each frame with a two-dimensional Gaussian profile and the achieved resolution was in the order of nanometers. Using these records we determined the power spectral density which was fitted by a Lorentzian function^29^,^30^,^31^. The corner frequency *f_ci_* was the key fitted parameter which determines the stiffness of the optical trap along axis *i*: 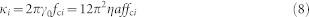
where *γ*_0_ = 6*πηaf* denotes hydrodynamic drag coefficient, *η* is dynamic viscosity and *f* is a particle shape correction factor^60^ considering *a* as the radius of sphere of the same volume. Knowledge of both *η* and *f* is crucial for proper determination of the trap stiffness, however, *η* depends on temperature and *f* on the NP orientation in the optical trap.

We used Comsol Multiphysics to calculate the total amount of laser power *P* absorbed by Au triangular nanoprism (aspect ratio 0.15, corresponding sphere diameter 100–250 nm) and by decahedral particle (corresponding sphere diameter 100 nm). We considered the NPs to be located in their stable axial location and stable orientation, *i.e.* triangular particle is oriented perpendicularly to the beam polarization while decahedral particle is parallel to beam polarization. Laser power was taken to be 100 mW, corresponding to the experimental value. We used the absorbed power to calculate the temperature increase of the NP surface assuming stationary heat transfer from spherical NP of radius *a*^13^: 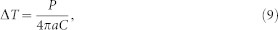
where *C* = 0.6 W/(Km) is the thermal conductivity of water. The temperature increase is approximately 50°C for NPs of diameter smaller than 200 nm and 80°C for NPs of diameter equal to 250 nm, respectively. In the case of decahedral particle we obtained temperature increase 10°C for corresponding sphere diameter of 100 nm, however no stable position existed for larger decahedral NPs. Higher temperature increases of the triangular nanoprisms are caused by the fact that stable NPs position is located closer to the beam focus than decahedral NPs. The trapped NP heats the medium and thus the medium viscosity decreases^13^, *e.g.* increase of water temperature by 20 or 50°C reduces viscosity to 65% or 40% of that at the room temperature, respectively. Correspondingly, the trap stiffness decreases with decreased medium viscosity.

The shape of investigated NPs is close to spheroids and therefore we can use with advantage the analytical formulas for the drag coefficients derived by Perrin^60^. For oblate spheroids, the following correction factors to the drag coefficients can be derived: 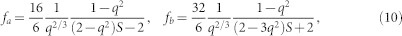
*q* is the ratio of one of the longer semi-axes *b_e_* to the shorter semi-axis *a_e_* (*q* = *b_e_*/*a_e_*) and 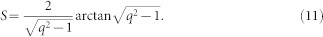
*f_a_* and *f_b_* corresponds to the motion of spheroid along the shorter semi-axis *a_e_* and any of the longer semi-axes *b_e_*, respectively. Considering the thin triangular nanoprism, we obtain for its aspect ratio the correction factors *f_a_* = 1.61 and *f_b_* = 1.20, *i.e.* an increase of *κ_x_* by 61% and *κ_z_* by 20% for a stably oriented NP. Since the increase of viscosity with temperature and the influence of the shape correction factors go against each other, we do not expect significant deviations from the values presented in Fig. 7 within the estimated standard deviations.

## Author Contributions

O.B. and P.Z. developed the presented method and supervised the project. O.B., M.Š. and P.Z. wrote the manuscript. O.B. performed all the experiments and subsequent data analysis. O.B., M.Š., J.T., L.C. and V.K. performed computer simulations. A.P., Z.P. and F.M. performed scanning electron microscopy of metal nanoparticles.
